# DNA damage and repair in plants – from models to crops

**DOI:** 10.3389/fpls.2015.00885

**Published:** 2015-10-23

**Authors:** Vasilissa Manova, Damian Gruszka

**Affiliations:** ^1^Department of Molecular Genetics, Institute of Plant Physiology and Genetics, Bulgarian Academy of SciencesSofia; ^2^Department of Genetics, Faculty of Biology and Environment Protection, University of SilesiaKatowice, Poland

**Keywords:** *Arabidopsis*, crop plants, DNA damage, DNA repair, mutagenesis

## Abstract

The genomic integrity of every organism is constantly challenged by endogenous and exogenous DNA-damaging factors. Mutagenic agents cause reduced stability of plant genome and have a deleterious effect on development, and in the case of crop species lead to yield reduction. It is crucial for all organisms, including plants, to develop efficient mechanisms for maintenance of the genome integrity. DNA repair processes have been characterized in bacterial, fungal, and mammalian model systems. The description of these processes in plants, in contrast, was initiated relatively recently and has been focused largely on the model plant *Arabidopsis thaliana*. Consequently, our knowledge about DNA repair in plant genomes - particularly in the genomes of crop plants - is by far more limited. However, the relatively small size of the *Arabidopsis* genome, its rapid life cycle and availability of various transformation methods make this species an attractive model for the study of eukaryotic DNA repair mechanisms and mutagenesis. Moreover, abnormalities in DNA repair which proved to be lethal for animal models are tolerated in plant genomes, although sensitivity to DNA damaging agents is retained. Due to the high conservation of DNA repair processes and factors mediating them among eukaryotes, genes and proteins that have been identified in model species may serve to identify homologous sequences in other species, including crop plants, in which these mechanisms are poorly understood. Crop breeding programs have provided remarkable advances in food quality and yield over the last century. Although the human population is predicted to “peak” by 2050, further advances in yield will be required to feed this population. Breeding requires genetic diversity. The biological impact of any mutagenic agent used for the creation of genetic diversity depends on the chemical nature of the induced lesions and on the efficiency and accuracy of their repair. More recent targeted mutagenesis procedures also depend on host repair processes, with different pathways yielding different products. Enhanced understanding of DNA repair processes in plants will inform and accelerate the engineering of crop genomes via both traditional and targeted approaches.

## Introduction

Cellular DNA of living organisms normally suffers damage which may arise endogenously or can be induced by a variety of external genotoxins including ultraviolet light, ionizing radiation, and chemical mutagens. The most frequently encountered injuries to the DNA- often induced through inevitable errors of internal metabolism- are modifications to nucleotides, intra- or inter-strand cross-links, and breaks of the phosphodiester bonds. If damaged DNA is not repaired it may have difficulties in being properly organized, replicated, or transcribed. The impairment of such essential molecular processes affects cellular functionality and may disturb the normal development of the whole organism (Britt, 1996; Polyn et al., 2015*)*. Plants are particularly vulnerable to the DNA damaging factors present ubiquitously in the air, soil, and water. Hence, they have evolved a complex network of mechanisms of DNA damage detection and repair dedicated to ensure their genomic stability through removal of the DNA lesions and reconstitution of the original genetic information (Bray and West, 2005; Yoshiyama et al., 2013). An intrinsic feature of certain DNA repair pathways is that they are not error-free, leading to potentially transmissible mutational alterations. The error-prone nature of some DNA repair mechanisms, however, increases the genetic diversity and variability of the populations, thus contributing to the evolution of plant genomes (Schuermann et al., 2005). Chemical or radiation-induced mutagenesis has been a powerful tool for creation and improvement of economically important crop varieties (Parry et al., 2009; Forster and Shu, 2012). The mutations occurring in the plant genome after particular mutagenic treatment are determined by both the spectrum of lesions generated by the mutagen and the specificity and efficiency of DNA repair pathways involved. Therefore, our understanding of DNA repair mechanisms and their regulation in plants is an essential requirement for the effective utilization of mutation technologies in future crop improvement.

It is generally accepted that the choice of a repair pathway and its action is primarily dependent on the type of the cell, its proliferation status, cell cycle stage, as well as on the type of the lesion and its genomic context (Britt, 1999). Plants do not choose where they live and cannot escape unfavorable environmental impacts. Therefore, they need strictly controlled but flexible DNA repair mechanisms responsive to the changing environment. Indeed, common external factors such as light regimes, temperature or water availability were shown to dictate the specific activation and efficiency of certain DNA repair pathways, such as recombination or photorepair in various plants (Li et al., 2002; Boyko et al., 2005; Chang et al., 2008). Rapidly dividing and differentiated cells of different plant organs do not equally utilize the whole available repertoire of DNA repair mechanisms (Kimura et al., 2004; Boyko et al., 2006; Yang et al., 2010). Moreover, the capability of plants to maintain their genomic integrity was shown to decrease with plant age mainly due to a reduction in the efficiency and relative contribution of the employed DNA repair pathways (Golubov et al., 2010).

With some exceptions plants have been shown to possess all common DNA repair mechanisms which have been initially described to a greater extent in the other eukaryotic systems, such as yeast and mammals (Britt, 2002). Photoreactivation of UV-induced DNA damage is one of the primary DNA repair mechanisms needed by plants on a daily basis because of their inherent necessity and exposure to solar light. The two classical forms of excision repair, base (BER) and nucleotide (NER), often regarded as “dark repair”, are also available for the plant genome suffering various types of DNA lesions (Rastogi et al., 2010). MMR has been implicated in the removal of incorrectly paired nucleotides and the UV-induced photolesions from the genome of higher plants (Culligan and Hays, 2000; Lario et al., 2011). The main DNA double-strand break repair pathways – HR and NHEJ have been shown to be essential in plants for the preservation of their genetic stability (Puchta and Hohn, 1996; Waterworth et al., 2011). Some of the repair mechanisms as photoreactivation are highly specialized for a particular damage, however, others, like excision or recombination pathways may deal with a variety of lesions (Ries et al., 2000a).

Significant progress in elucidation of DNA damage repair in higher plants has been made mainly utilizing the small dicot *Arabidopsis thaliana* as a model (Hays, 2002). The isolation and characterization of the first plant DNA repair genes involved in the photorepair, excision repair, HR and NHEJ have been initially based on the homologous sequence information available from other organisms (Batschauer, 1993; Britt et al., 1993; Santerre and Britt, 1994; Ahmad et al., 1997; Jiang et al., 1997a; Doutriaux et al., 1998; Garcia et al., 2000; Hartung et al., 2000; Liu et al., 2000; Osakabe et al., 2002; Tamura et al., 2002; West et al., 2002, etc.). During the last decade significant progress has also been made in the molecular characterization of the repair pathways and genes mediating these processes in important crop plants such as rice, spinach, cucumber, tomato, wheat, barley, etc. The headlong progress of molecular technologies has expanded the number of sequenced crop genomes and thus contributed to the advancements made in the field of plant DNA repair as well (Singh et al., 2010; Kim et al., 2015). In addition to *Arabidopsis*, rice is the other higher plant with relatively well characterized DNA repair mechanisms with respect to the influence of various developmental and environmental factors on their activation and efficiency, as well as regarding the identification and regulation of genes involved in the DNA repair and protection mechanisms (Ueda and Nakamura, 2011). The sequence of the rice genome has been useful for the efficient identification of orthologous genes, regulatory regions and gene functions in other cereals (Goff et al., 2002). For example, currently identified barley and wheat genes display ∼80% homology, at the nucleotide level, to their rice counterparts thus substantiating the usefulness of rice homologous sequences for identification of the DNA repair-associated genes in other monocots. The intensive research performed on *Arabidopsis* and rice has enormously increased the current knowledge on the molecular nature and regulation of DNA damage and repair mechanisms in plants. However, such studies should be expanded to include a larger number of model and crop species if we want to have a clearer picture of the capacity of plant genomes to overcome the biological impacts of different genotoxins and to adapt to the changing environmental stress conditions.

## DNA Damage Induced by Endogenous and Exogenous Factors

**FIGURE 1:**
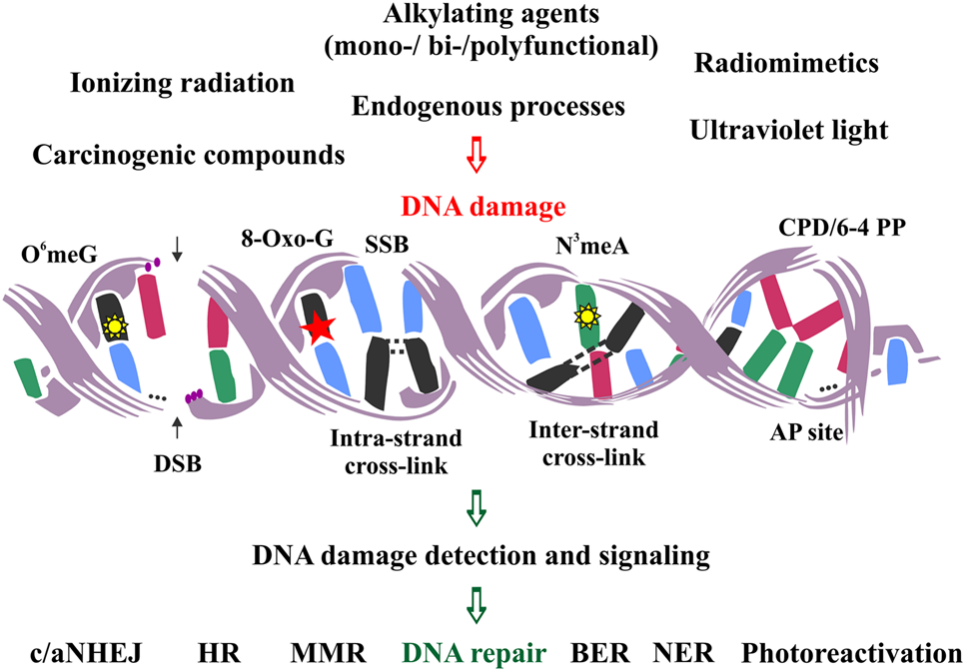
**Schematic representation of the major DNA lesions induced by various external and endogenous factors, and the types of DNA repair mechanisms employed to remove them from the eukaryotic genome**.

DNA lesions are divided into two main categories: single- and double-stranded. The first category is comprised of lesions disturbing only one DNA strand, such as oxidized or alkylated base damage, base loss, DNA adducts, intra-strand cross-links, DNA photoproducts and single-strand DNA breaks (SSBs). The second category includes lesions affecting both DNA strands, such as inter-strand cross-links and double-strand DNA breaks (DSBs), the latter being the most severe type of DNA damage in the eukaryotic genome (**Figure 1**).

### Endogenously Arising DNA Lesions

A major source of endogenous DNA lesions is the intracellular metabolism which increases the concentration of free radicals in the environment surrounding the DNA; in plants, ROS are especially ubiquitous in the chloroplasts and mitochondria (Sharma et al., 2012). AP sites may arise by spontaneous hydrolysis of the N-glycoside bond or as intermediates resulting from the repair of deaminated, alkylated or oxidized bases (Cooke et al., 2003; Tuteja et al., 2009). Moreover, all DNA-associated processes involved in the transmission, expression and maintenance of genetic information have the potential to cause SSB or DSB in DNA (Bessho, 2003; Edlinger and Schlögelhofer, 2011; Montecucco and Biamonti, 2013).

### DNA Lesions Caused by Exogenous Damaging Factors

#### Alkylating Agents

Monofunctional alkylators such as MMS and EMS are the chemical agents most widely utilized to obtain mutagenized plants aimed at both crop improvement and reverse genetics studies (Till et al., 2003; Natarajan, 2005). Alkylating agents methylate the DNA bases, mainly at their O- and N-positions generating small base damage as O^6^-methylguanine (O^6^-meG), N^7^-methylguanine or N^3^-methyladenine (Shrivastav et al., 2010). Bi- and polyfunctional alkylating agents as well as many carcinogenic compounds form intra-strand cross links between adjacent guanines or bulky adducts to nucleotides which significantly distort the conformation of the DNA molecule. Psoralens and mitomycin C can also induce inter-strand cross-links connecting the two opposite DNA strands thus effectively blocking the replication and transcription machineries (De Silva et al., 2000).

#### Ionizing Radiation

Ionizing radiation in the form of gamma- and X-rays as well as ion-beams is another commonly employed DNA damaging agent with high mutagenic potential in plants (van Harten, 1998). It produces a large number of lesions through a direct ionization of the DNA molecule or indirectly via an initial interaction with water resulting in the subsequent radiolysis and production of highly reactive species, such as hydroxyl radicals (OH^•^), free electrons (e-) and hydrogen radicals (H^•^) (Alpen, 1998). In the case of a direct event the high-energy deposition of IR generates DSB which leads to DNA fragmentation. IR-induced DSBs frequently have modified termini such as 5′hydroxyl, 3′phosphate, and 3′glycolate, which need processing to make them compatible for ligation (Schärer, 2003). Oxidation products (8-oxoguanine, thymine glycols, etc.), base loss (AP lesion of “regular” or “oxidized types”) as well as SSB are amongst the lesions generated via secondary DNA ionization. Moreover, IR induces multiple damaged sites representing two or more closely localized lesions on the same or the opposite DNA strands (Shikazono et al., 2009), which usually involve SSB with damaged termini accompanied by modified or damaged bases and deoxyribose moieties with a significantly higher frequency than the frank DSB (Sutherland et al., 2000). Recent research shows that such a cluster might transform to DSB as a result of excision repair, but this probability depends on the local chromatin environment (Cannan et al., 2014). It was generally thought that IR-induced DSBs are spread rather randomly in the genome; however, an accumulating body of evidence reveals the influence of chromatin organization and nuclear matrix proteins on DSB distribution (Lavelle and Foray, 2014).

#### Radiomimetic Agents and REs

Double-strand breaks are also produced by a variety of radiomimetic agents, so-called because of their ability to act on the DNA by mimicking the effects of IR. The anticancer drug BLM which is frequently utilized in the studies of DSB formation and repair in mammalian cells has been shown to effectively generate DSB in many plant systems as well. That is why numerous DNA repair assays based on the BLM action have been introduced in a variety of plant species (West et al., 2002; Manova et al., 2006; Georgieva and Stoilov, 2008; Kozak et al., 2009; Stolarek et al., 2015a,b). BLMs are a family of glycopeptides which cannot diffuse freely through the cellular membranes due to their hydrophilic properties, but are transferred into the cell by a receptor-mediated endocytosis (Chen and Stubbe, 2005). A single BLM molecule can cause breakage in the double-stranded DNA thus generating blunt DSB ends or termini with non-complementary single-base extensions. In addition, abasic sites with closely opposed SSB can also result from the BLM action in a frequency exceeding that expected by the coincidence of two independent damaging events. Oxygen levels of the microenvironment could modify the type of BLM-generated DNA damage – a lack of O_2_ favors the formation of AP site, whereas the presence of O_2_ facilitates formation of a DSB with 5′-phosphate and 3′-phosphoglycolate ends (Povirk and Finley Austin, 1991). The action of BLM is modulated by the local nucleosome structure and higher-order chromatin organization (Smith et al., 1994), leading to the preferential DNA breakage in the linker DNA of mammalian and plant cells (Kuo, 1981; Manova et al., 2006).

Restriction endonucleases produce only DSBs, which may be of “blunt” or “cohesive” type, but are always readily ligatable with 5′-phosphate and 3′-hydroxyl groups (Bryant, 1990). REs have a high clastogenic activity on the genomes of mammals and plants (Obe et al., 1995; Stoilov et al., 2000; Manova and Stoilov, 2003). The unique selection ability of rare-cutting endonucleases has been used to develop highly specialized transgenic systems in order to monitor somatic HR in various plants such as *Arabidopsis*, tobacco and rice (Puchta et al., 1995; Puchta and Hohn, 2012). The more recently developed chimeric nucleases designed to target particular genomic locations and introduce DSB at specific DNA sequences have the potential to broaden the studies of DSB rejoining in plant genomes (*see later*).

#### Ultraviolet Radiation

Ultraviolet radiation, being a component of sunlight, is the most common genome-damaging agent ubiquitously found on earth (Britt, 2004). It belongs to the electromagnetic radiation spectrum with wavelengths ranging from 100 to 400 nm. There are three ranges of UV radiation: short UV-C (100–280 nm), which is the most harmful for the genetic material as it is directly absorbed by the DNA, middle-range UV-B (280–315 nm) which is the main DNA damaging component of the solar light, and long wavelength UV-A (315–400 nm).

UV light generates two major types of lesions in DNA – CPDs and 6-4 PPs, whose relative proportion and non-random distribution within the eukaryotic genome depends on the sequence composition and chromatin structure (Pfeifer, 1997; Kwon and Smerdon, 2005; Law et al., 2013). In plants the CPDs may account for up to 90% of all pyrimidine dimers induced upon exposure to UV-B (Dany et al., 2001). In the case of CPD the covalent bonds are formed between the C-5 and C-6 carbon atoms of the adjacent pyrimidine bases, usually between TpT and less frequently between TpC and CpC sequences (Durbeej and Eriksson, 2003). The 6-4 PPs are typically formed between the carbon atoms at C-6 and C-4 positions of an adjacent TpC dinucleotide (Pfeifer et al., 1991). The presence of CPDs has the potential to block the transcribing complexes thus completely altering the relative expression pattern of genes (Tornaletti et al., 1999). During replication, however, dimers can be bypassed by specialized translesion DNA polymerases which increase the cellular tolerance to UV damage, also in plants (Britt, 1995; Curtis and Hays, 2011; Nakagawa et al., 2011).

UV radiation may also induce oxidative DNA damage, mediated predominantly, but not exclusively, by endogenous photosensitizers that generate free radicals upon their activation. The genotoxic effects of oxidative DNA damage were clearly demonstrated in mammalian cells (Roldán-Arjona et al., 2002). Although rare, there are studies showing the presence of UV-induced oxidative DNA lesions in plants (Watanabe et al., 2006). As in plants pyrimidine dimers are primarily repaired by photoreactivation, it might be speculated that oxidative DNA damage, known to be eliminated by the error-prone excision repair, could also contribute to the UV-associated mutagenicity and plant genomic instability.

## DNA Repair Pathways in Model and Crop Plants – Nature and Efficiency, Genetic Control, and Agricultural Importance

### Photoreactivation

**FIGURE 2:**
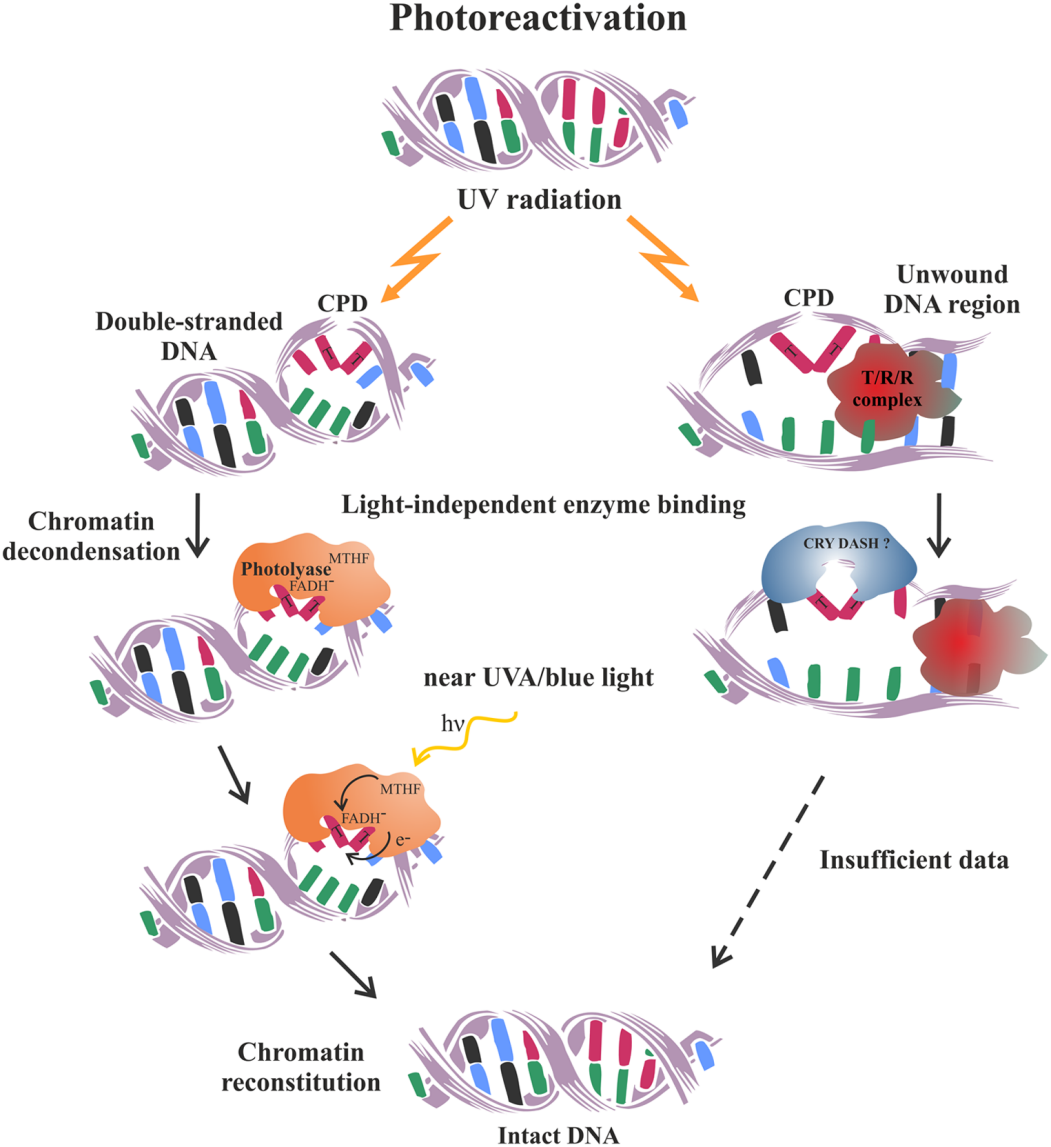
**Schematic representation of the photoreactivation mechanism utilized by plants to repair UV-induced pyrimidine dimers**. On the left is schematized the classical well characterized process of CPD photoreactivation in double-stranded DNA. On the right is presented a hypothetical mode of CPD photoreactivation, which might operate within the open DNA regions generated during transcription, replication and repair processes. It is thought to bemediated by the DASH chryptochromes, based on their ability to photoreactivate CPD in single-stranded DNA (Pokorny et al., 2008). Future research is needed to prove or reject such an intriguing concept. T/R/R complex – transcription, replication or repair complex.

Photoreactivation is a rare example of a simple and error-free pathway for the reversal, rather than the removal of DNA damage. It is performed by a single, lesion-specific enzyme called photolyase. It is thought to be the first DNA repair pathway to have evolved in early life forms, which is still being maintained in such various organisms as bacteria, yeast, plants, and animals, but has been evolutionarily lost by the placental mammals (Lucas-Lledó and Lynch, 2009). Two different types of photolyase enzymes have been established in plants which are specialized to reverse selectively the 6-4 photoproducts (6-4 photolyase type) or the CPD (class II photolyase). Photolyases bind to their specific damage-substrate within the double-stranded DNA in a light-independent manner. However, in order to get energy for correcting the lesion they need to be excited by photons from the blue or near UV-A spectrum (Brettel and Byrdin, 2010) (**Figure 2**). Subsequently, the electron is transferred from the CPD photolyase chromophore to the lesion and after splitting the dimer’s covalent bonds, it is returned back to restore the catalytically active state of the cofactor (Thiagarajan et al., 2011). The exact chemistry of the photorepair reactions differ between the two photolyase types, however, the final products are monomerized pyrimidine bases and unchanged nucleotide sequence (Yi and He, 2013). The error-free nature of photoreactivation makes it the preferable and most effective mechanism utilized by plants to quickly reduce the negative effects of DNA photodimers generated upon their normal exposure to solar radiation (Dany et al., 2001; Britt, 2004). However, inability of the cell to photorepair may lead to a switch in the transcriptional response of UV stressed plants activating the completely different DNA repair pathway such as HR (Molinier et al., 2005).

Based on the UV-B sensitive *Arabidopsis* mutants deficient in either CPD or 6-4 PP repair two different plant photorepair genes have been identified – *PHR1* coding for the CPD photolyase and *UVR3* which encodes the 6-4 photolyase (Britt et al., 1993; Jiang et al., 1997b; Landry et al., 1997; Nakajima et al., 1998). Effective light-dependent repair of DNA photolesions has been demonstrated in all plants investigated so far and the lesion-specific photolyases identified in *Arabidopsis* (Pang and Hays, 1991; Chen et al., 1994; Ahmad et al., 1997) have also been cloned and/or reported to operate in a variety of other plant species like maize (Stapleton et al., 1997), wheat (Taylor et al., 1996), white mustard (Batschauer, 1993), cucumber (Takeuchi et al., 1998), spinach (Yoshihara et al., 2005), alfalfa (Quaite et al., 1994), rice (Hirouchi et al., 2003), soybean (Yamamoto et al., 2008), barley (Manova et al., 2009), etc. In view of the importance of the photorepair mechanism for plant growth and development under UV exposure it is not surprising that the CPD photolyase has become one of the most intensively studied DNA repair genes in higher plants. Photolyases belong to a special category of proteins, the photolyase/cryptochrome family, containing flavin as a cofactor and, depending on the species, a “second” chromophore acting as a photoantenna (Thompson and Sancar, 2002). Plant CPD photolyases analyzed so far, particularly the *Arabidopsis* and rice enzymes, show similar chromophore compositions, consisting of both a reduced FADH and a pterin-like cofactor (Waterworth et al., 2002; Teranishi et al., 2008).

The cryptochromes work mainly as photoreceptors regulating plant development as they do not possess the standard photorepair activity characteristic of the photolyase proteins. More recently, it has become evident that a special subclass of these flavoproteins actually has DNA-binding and photorepair activity, but only on single-stranded and/or loop DNA structures. These intriguing observations have inspired the hypothesis that DASH-type cryptochromes might be involved in the repair of the locally unwound DNA regions generated by the active matrix processes such as replication, transcription, and DNA repair (Selby and Sancar, 2006; Pokorny et al., 2008).

Chromatin organization affects not only the formation, but also the repair of UV-induced pyrimidine dimers. In yeast the access of photolyase proteins to the UV-damaged DNA was inhibited in the compacted chromatin, whereas the local nucleosome unpacking and repositioning facilitated photoreactivation (Thoma, 2005). It has been recently shown that histone-binding proteins of the ASF group are essential for CPD repair in *Arabidopsis* specifically under light conditions (Lario et al., 2013). Moreover, chromatin factors and histone acetylation have been important prerequisites for UV damage removal in both *Arabidopsis* and maize, suggesting an important role of chromatin restructuring for the effective photorepair process also in plants (Campi et al., 2012).

Currently the data obtained in different plant species concerning the distribution of photolyase proteins and their activities within the intracellular compartments are rather controversial. A number of studies have shown that CPD photolyase is present exclusively in the nucleus thus implying that extranuclear DNA in plant mitochondria and chloroplasts cannot be photoreactivated. Indeed, fractionated extracts from spinach chloroplasts were found to be free of the photolyase activity otherwise contained in whole leave preparations (Hada et al., 2000). The lack of CPD repair from spinach organelles confirmed the earlier findings showing that young *Arabidopsis* seedlings were able to remove CPD only from the nuclear genome, but not from the chloroplast and mitochondrial DNA sequences (Chen et al., 1996). In line with the idea, in *Arabidopsis* it was demonstrated that the CPD photolyase protein is transported only into the nucleus, but not in the chloroplasts (Kaiser et al., 2009).

On the other hand, light-dependent repair with varying efficiency was found in the individual soybean and maize genes localized not only in the nucleus but also in the chloroplast and mitochondrial genomes (Cannon et al., 1995; Stapleton et al., 1997). The fully developed *Arabidopsis* leaves also repaired CPDs and 6-4 PPs in the nuclear and chloroplast DNA upon prolonged blue light exposure and restored the replication of both nuclear and organellar genomes. These observations illustrate that young and mature plants may differ in their DNA repair capacity, which in turn may affect their overall tolerance to UV stress (Draper and Hays, 2000). Moreover, data obtained in rice reveal that CPD photolyase is localized not only in the nucleus, but is also active in the mitochondria and plastids. The presence of CPD photolyase in the organelles allows the rice leaf cells to employ photoreactivation to restore the integrity of extranuclear DNA after UV-B exposure (Takahashi et al., 2011). Phosphorylation, particularly at serine-7, was found to be an important modification of rice CPD photolyase, regulating protein translocation within the cellular compartments (Teranishi et al., 2013). In rice nuclei and chloroplasts the photolyase is mainly present in its phosphorylated form, whereas in the mitochondria the major part of the protein is unphosphorylated. Recently, unique sequences targeting rice CPD photolyase to the mitochondria have been identified in the C-terminal region of the protein and validated as functionally important (Takahashi et al., 2014). Therefore, further studies will reveal whether the access of plant photolyases to the organellar genomes is species-specific, or whether it depends on the developmental stage of the plant, as well as on the organ or tissue affected, or there are other factors at the molecular level which might control the presence and activity of plant photolyase proteins within the nuclear and extranuclear plant genomes.

### Photorepair and its Impact on Agriculture

The inhibitory effect of the UV-component of natural light on *Arabidopsis* growth affected plant height and the rosette diameter in both wild-type and the DNA repair mutants, and those defective in the PHR1 were found to be most sensitive to the daily UV-B exposure (Britt and Fiscus, 2003). Regarding crop plants, intensive research has focused on the variations of CPD photorepair efficiency as a primary determinant of UV-B tolerance affecting the development of individual rice cultivars. The agriculturally valuable and therefore widely cultivated variety Norin1 was more sensitive to UV-B radiation compared to the Sasanishiki cultivar. Notably, the lower capacity of plant leaves to repair CPDs was the primary cause of the reduced productivity and growth of Norin1 (Hidema et al., 1997). It was shown that even a single nucleotide variation changing Gln-126 (in Sasanishiki) to Arg (in Norin 1) may generate structural alterations in the photolyase protein, thus affecting the stability of the CPD-photolyase complex. The resulting decrease in the repair activity of the enzyme thus explains the significant differences in the UV resistance between the two cultivars (Hidema et al., 2000; Teranishi et al., 2004). Moreover, spontaneously occurring variations in other crucial domains of CPD photolyase protein have changed the photorepair ability of many cultivated and wild rice genotypes, thus creating differences in their UV-B sensitivity finally manifested as reduced growth and productivity (Hidema et al., 2005; Iwamatsu et al., 2008). Combined these studies demonstrate that UV tolerance is a vital characteristic of the contemporary plant cultivars and DNA repair, and photolyase in particular, might be an important target for modification to improve economically important crops in modern breeding programs (Hitomi et al., 2012).

The capability of plants to photoreactivate UV-induced pyrimidine dimers depends also on the available amounts of the photolyase protein within the damaged cell. The first indication of the influence of light environment on the transcriptional regulation of plant photolyase genes came from the observation that the etiolated plants had a lower capacity to repair CPDs than the de-etiolated. On the other hand, the 6-4 photoproducts were repaired very efficiently not only in the green, but also in the etiolated *Arabidopsis* plantlets. A lack of gene transcripts and CPD photolyase activity in the dark-grown seedlings has been reported in several plants such as mustard, *Arabidopsis* and spinach (Batschauer, 1993; Chen et al., 1994; Yoshihara et al., 2005). Within the first hours of light exposure the expression of *PHR1* and the accumulation of functional protein increased drastically. The light-dependent regulation of *PHR1* possibly underlined the diurnal fluctuations of the photorepair activity measured in cucumber leaves (Takahashi et al., 2002). The continuous light exposure, however, may finally reduce the CPD photolyase transcript levels if not supplemented with a UV-B component (Waterworth et al., 2002). Intense UV-B radiation also enhanced the expression of *PHR1* gene in *Arabidopsis* and cucumber, but at the same time inhibited the activity of the protein in the latter (Ries et al., 2000b; Takeuchi et al., 2007). In fact, etiolated plant tissues may contain certain amounts of CPD photolyase transcripts and activity allowing initiation of CPD repair immediately upon their exposure to visible light (Takeuchi et al., 2007; Castells et al., 2010). In *Arabidopsis*, the dark inhibition of photorepair gene expression is maintained through the photomorphogenic repressors AtDET1 and AtCOP1 until their negative action is alleviated during de-etiolation. However, the transcriptional induction of photolyase genes, both *AtPHR1* and *AtUVR3*, is further stimulated by the HY5 and HYH transcription factors (Castells et al., 2010). A key player of plant response to UV-B is the recently identified UV-B specific photoreceptor AtUVR8 which manages a downstream signaling cascade involving COP1 and HY5, which in turn, control the expression of many genes related to both plant UV-protection and photomorphogenic response (Tilbrook et al., 2013). In line, the *Atuvr8-2* mutant is unable to induce *PHR1* gene expression, which is normally found in the wild-type plants exposed to low UV-B levels (Brown and Jenkins, 2008). Another report also shows that under normal conditions *AtPHR1* is targeted by the transcriptional repressor E2Fe/DEL1 involved in the regulation of the endoreduplication potential of the cell, but this suppression is alleviated upon UV-B exposure allowing the induction of *AtPHR1* (Radziejwoski et al., 2011).

The above results demonstrate that the manipulation of plant CPD photolyase at the transcriptional level might be a promising approach for the generation of UV resistant plants. Indeed, transgenic *Arabidopsis* lines overexpressing CPD photolyase showed an improved repair capacity and enhanced UV tolerance (Kaiser et al., 2009). On the other hand, by silencing the same gene Yoshihara et al. (2008) were able not only to decrease the UV-B tolerance of the knocked-down plants, but also to increase the rate of transitions and frameshift mutations in the *Arabidopsis* genome. Hence, photolyase genes seem valuable for the modern agriculture for at least two reasons – the first is to improve the UV tolerance and productivity of the economically important cultivars, and the second might be to aid mutagenesis studies by enhancing the efficiency of mutation induction in the plant genome.

### Direct Repair by DNA Alkyltransferases

Another form of a direct DNA damage repair is the specific processing of alkylated bases executed by the enzyme called AGT. The repair mechanism includes direct transfer of the alkyl group from the lesion to the cysteine residue located in the active center of the enzyme; as the reaction is irreversible the inactivated protein undergoes further proteasome degradation. AGT is a compact single chain protein which scans the genome and provides fast repair of the lesions due to its efficient binding into the minor groove of DNA (Pegg, 2011). It is believed that the enzyme can target itself to the sites of active transcription, where it ensures the error-free repair of O^6^-meG especially in the transcriptionally active regions (Ali et al., 1998). The non-repaired O^6^-meG can be bypassed during both replication and transcription giving rise to transition mutations and altered mRNA molecules, respectively (Iyama and Wilson, 2013). In human cells, AGT functioning is essential to prevent the accumulation of altered transcripts and mutant proteins (Burns et al., 2010). AGTs are ubiquitously present from bacteria to humans; the only organisms lacking this repair enzyme are fission yeast and plants (Pegg, 2011). Indeed, the search for AGT homologs in both model and crop plants has been largely unsuccessful so far (Costa et al., 2001). In plants O^6^-meG is a preclastogenic lesion associated with the formation of chromosomal aberrations (Baranczewski et al., 1997a). In *Vicia faba* root tips, however, the reduction of chromatid aberration frequency has been correlated with an effective removal of O^6^-meG implying the presence of rapid adaptive mechanisms against this lesion in plant genome (Baranczewski et al., 1997b). It is therefore quite probable that other DNA repair pathways efficiently substitute the lack of AGT activity in plants and recent research implicates BER as such a possible candidate.

### Base Excision Repair

Base excision repair is active on a wide range of lesions, such as damaged or modified bases as well as naturally occurring AP sites. It is initiated by damage-specific DNA glycosylases which cleave the N-glycosidic bond and remove the affected base generating an abasic site. AP endonuclease or AP lyase activities are further necessary for processing of the resultant AP site. Subsequently the repair reaction may proceed by either a “short” or a “long” patch mechanism depending on the type of lesion and the enzyme engaged. In mammalian cells, the “short” mode of BER employs the activity of DNA polymerase β (polβ), XRCC1 and Ligase III which accomplish the repair by excision of only one nucleotide. The ”long-patch” BER involves the removal of up to 10 nucleotides surrounding the lesion and relies on the activity of polymerase complex δ/ε-PCNA-FEN1. It is important to note that single strand DNA breaks are unavoidable intermediates during BER, and as such they may become substrates of other repair mechanisms such as NER and recombination repair (Memisoglu and Samson, 2000).

Up to now, several lesion-specific DNA glycosylases have been identified in plants. *Arabidopsis* 3-methyladenine-DNA glycosylase, the first cloned plant DNA repair gene, was shown to remove the alkylated DNA lesions induced by MMS treatment (Santerre and Britt, 1994). In whole-cell extracts isolated from *Arabidopsis* the uracil containing DNA is repaired by the BER pathway employing enzymes from the uracil-DNA glycosylase family. Notably, both BER modes may occur after the initial incision steps despite the lack of polβ and Ligase III homologs in plants, and the repair reactions are finalized by the ligating activity of AtLIG1 (Córdoba-Cañero et al., 2009, 2011). In fact, *in vitro* monitoring of DNA repair reactions performed with cellular extracts isolated from *Arabidopsis* or other plants have been extremely useful to describe many of the structural and functional aspects of plant BER. Short-patch BER was revealed as an essential DNA repair pathway in plant mitochondria, at least for the removal of uracil, where uracil-DNA glycosylase activity was found associated predominantly with the organellar membrane in both the model and crop species (Boesch et al., 2009). In *A. thaliana* the AtFPG and AtOGG1 enzymes showed glycosylase/lyase activities to initiate the repair of oxidative lesion 8-oxoG and endogenous AP sites, and the generated intermediates later became substrates of the ZDP DNA 3′-phosphatase and ARP endonuclease. In line with their proposed role in the repair of oxidized bases, inactivation of the *AtFPG* and *AtOGG1* genes increased the level of oxidative DNA lesions in both the nuclear and mitochondrial genomes, whereas impairment of AtARP and AtZDP functions accelerated seed aging (Córdoba-Cañero et al., 2014). The putative AtPol λ has been implicated in the synthesis step of “long-patch” BER in *Arabidopsis*, based on the previously identified X-family DNA polymerase Pol λ from rice, although a contribution of yet unidentified plant DNA polymerases has not been excluded (Córdoba-Cañero et al., 2009; Uchiyama et al., 2009). As mentioned earlier, experimental evidences obtained so far show that LIG1 is the only ligase which can be unambiguously associated with both the “short” and the “long” patch BER in plants (Córdoba-Cañero et al., 2011).

Base excision repair is also implicated in the epigenetic control, as it is actively involved in the removal of 5-mC and its replacement with non-methylated C in eukaryotes, including plants. DNA methylation is an epigenetic modification associated with a compacted chromatin state which limits the accessibility of transcription factors or repair proteins to DNA. Therefore, improper BER functioning may prevent demethylation, and hence may affect gene regulation and activity of other DNA damage repair pathways by altering the expression of repair-associated genes. Several proteins have been recognized as being essential for different stages of BER-mediated demethylation processes in plants, such as the bifunctional DNA glycosylase/lyase ROS1, removing the methylated base and cutting the DNA strand, the DNA phosphatase ZDP involved in the processing of resulting intermediates as well as the *Arabidopsis* homolog AtXRCC1 which stimulates 3′-end cleaning and ligation steps (Martínez-Macías et al., 2013).

The *PCNA* homologs have been identified in *Arabidopsis* and various crop species, and some of them were found to possess a second functionally active copy of the gene. The plant PCNA proteins display many structural and functional similarities to those of other organisms supporting their involvement in the excision repair as well as in the replicative DNA synthesis pathways in plants (Strzalka and Ziemienowicz, 2011). Indeed, it was shown that the activities of both OsFEN1 and OsPolλ proteins in rice were enhanced by their interaction with OsPCNA, which is consistent with the active role of these enzymes in plant BER. Gene expression studies in rice coordinated BER activity with cell proliferation, as high levels of *OsPCNA*, *OsFEN1-a*, and *OsPol λ* transcripts were detected mainly in the developing rice tissues but not in the mature leaves, especially after DNA damaging treatment (Kimura et al., 2004; Uchiyama et al., 2004). In *Medicago truncatula*, upregulation of *MtOGG1* and *MtFPG* glycosylases was found during seed imbibition which coincided with water up-take and ROS production (Macovei et al., 2011). All of the above results imply that BER is extremely important for seed longevity as it helps the germinating embryo to repair oxidative DNA lesions accumulated during seed storage. Therefore, further knowledge on these mechanisms would help to improve preservation of the seeds and find new ways to maintain their germination potential. Although, BER activity in the mature plant organs might be less essential for the maintenance of DNA integrity, the involvement of BER in the establishment of epigenetic pattern of the genome implies important functioning of this pathway in transcriptional control at different stages of plant development.

### Nucleotide Excision Repair

**FIGURE 3:**
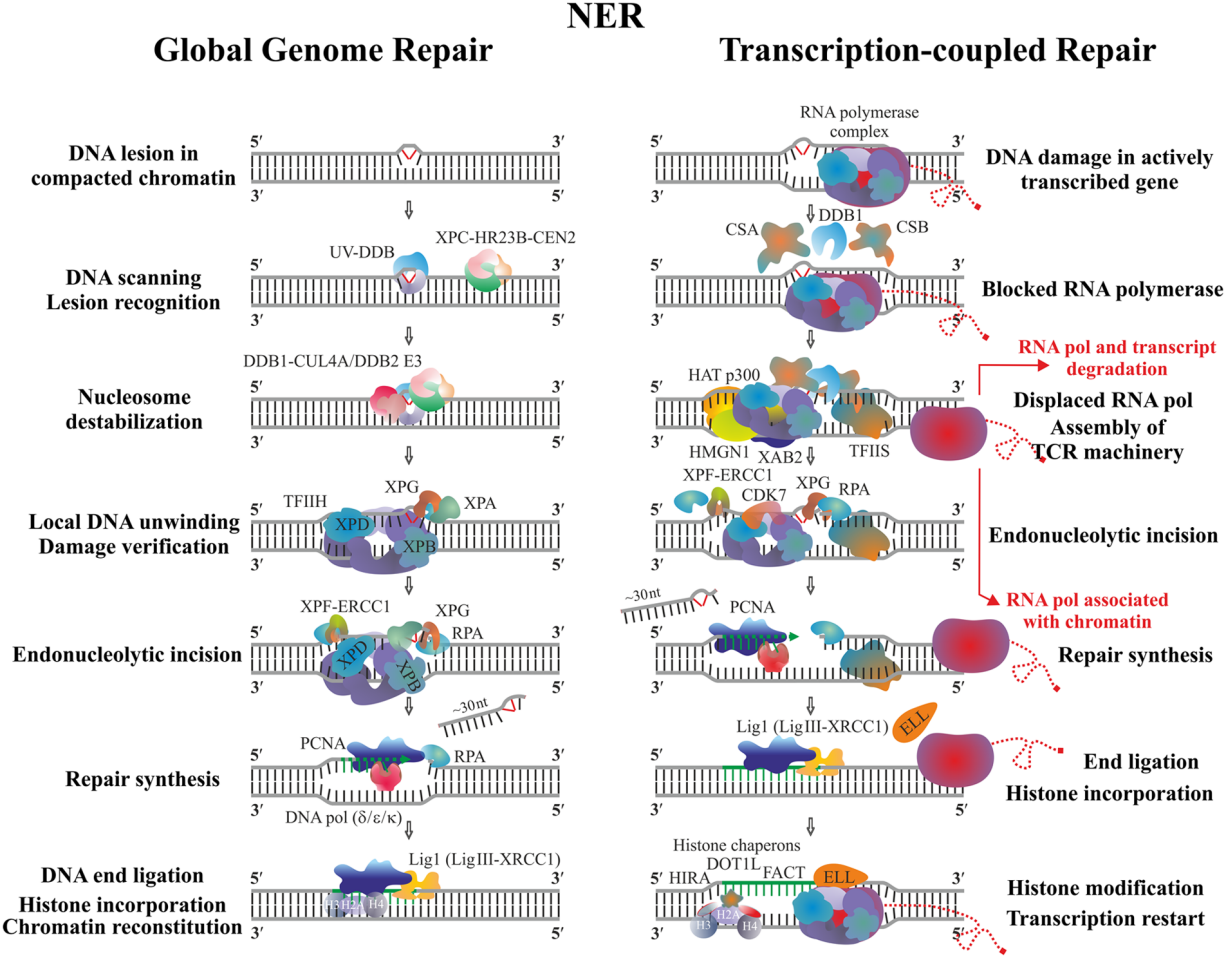
**Schematic representation of GGR and TCR in the eukaryotic cells**. Two main concepts exist regarding the fate of stalled RP. According to the first model, after completion of repair, the transcription continues from the point it has been impaired without degradation of RNA Pol II and its nascent transcript; a key role in this step is attributed to the elongation factor ELL which is thought to help the backtracked RNA pol II to release from chromatin as well as to attract additional, yet unidentified factors allowing transcription restart (Mourgues et al., 2013). Another possibility is that the stalled polymerase and its growing transcript are released from DNA and further degraded; the lesion is removed and transcription starts by new RNA pol II complexes from the beginning of the gene on an intact damage-free template (Sarasin and Stary, 2007).

Nucleotide excision repair is a general repair mechanism employed by both prokaryotic and eukaryotic cells to remove a variety of structurally different DNA lesions. The main substrates of NER are UV-induced photoproducts and other bulky DNA adducts that generate substantial conformational changes in DNA. The efficiency of NER varies within the different genomic locations and depends on the type of DNA lesion. Two distinct subpathways of NER exist – Global genomic repair (GGR) and Transcription-coupled repair (TCR). GGR is a whole genome repair pathway influenced by the chromatin structure and DNA-bound proteins, whereas TCR specifically accelerates the removal of transcription-blocking lesions from the template DNA strand of the highly expressed genes (Hanawalt, 2002). The two NER modes utilize specific factors but also share repair proteins and the main difference between them is in the system employed to sense the damage, whereas the later stages proceed in a similar way (**Figure 3**).

In animals GGR is initiated by the heterotrimeric complex Xeroderma Pigmentosum group C (XPC)/HR23B/Centrin2 which scans the genome and detects the damaged region based mainly on the distortion the particular lesion generates in the DNA double-helix, rather than on its chemical nature. The complex may be assisted by the heterodimer UV-DDB (composed of DDB1 and DDB2 subunits) helping to find lesions such as CPD, which only slightly alter the structure of DNA (Sugasawa, 2009). RPA, XPA, and XPG are then recruited along with the multiprotein complex TFIIH employed to verify the damage and identify which of the DNA strands is actually affected. TFIIH is also a component of the RNA polymerases I–III holoenzymes where it is crucial for transcription initiation and DNA unwinding during the early elongation step. Due to its complex dynamic structure and multifunctionality TFIIH is able to coordinate transcription and DNA repair, and may also relate these processes to the cell cycle control (Mydlikova et al., 2010). The recruitment of TFIIH during NER allows opening of the DNA duplex, which is performed by its key helicase subunits XPB and XPD, each of them bound to a different DNA strand. Their positioning within TFIIH determines the asymmetry and length of the opened DNA region; after verification the selected strand is nicked 5′ and 3′ to the lesion by XPF/ERCC1 and XPG nucleases, respectively (Fuss and Tainer, 2011). It was shown in mammalian cells that binding of RPA protects the single-stranded DNA end from degradation, whereas PCNA (either ubiquitinated or not) coordinates the repair synthesis and ligation steps performed selectively by DNA polymerases (δ, ε, or κ) and DNA ligase I or DNA ligase III/XRCC1 in a cell-cycle dependent manner (Moser et al., 2007; Ogi et al., 2010). A global relaxation of the native chromatin is required in order to provide access of repair complexes to the DNA lesion, which in turn has to be restored after the final ligation step (Palomera-Sanchez and Zurita, 2011). Chromatin modifications such as histone acetylation and ubiquitination seem to be tightly associated with the processivity and completion of NER. Local nucleosome destabilization might be mediated by the complex DDB1-CUL4A/(DDB2) E3, which is responsible for the ubiquitination not only of the NER factors DDB2 and XPC, but also of the histones H2A, H3, and H4 (Escargueil et al., 2008). Based on the latter observation, it has been suggested that the incorporated new histones may leave a kind of post-DNA repair imprints which could later affect epigenetic memory (Kamileri et al., 2012).

In higher eukaryotes the initial signal for TCR is emitted when the actively elongating RNA PolII complex stops on the transcribed strand due to the presence of damage. This is the reason why only this DNA strand is efficiently repaired whereas damage on the coding strand may persist for longer periods until being recognized by the GGR (Tornaletti, 2005). Key factors in TCR are CSA and CSB proteins (being components of different protein complexes) which bind the stalled RNAPolII. They, along with DDB1 initiate the assembly of other components of the TCR machinery such as HAT p300, HMGN1, XAB2, and TFIIS employed to displace the polymerase complex and modify chromatin, which results in exposure of the lesion to the common NER processing enzymes (Fousteri et al., 2006; Reed, 2011). In addition to the CDK7 kinase (essential during the processing step), novel TCR players have been identified as necessary for the continuation of gene transcription as soon as the template has been repaired. The elongation factor ELL is supposed to stimulate the dissociation of stalled RNA PolII from chromatin (Mourgues et al., 2013), whereas the proper chromatin environment, a prerequisite for successful post-repair restart of gene transcription, is ensured through modification of the newly assembled histones by specific histone chaperons such as HIRA, FACT, and DOT1L (Mandemaker et al., 2014).

Extensive studies in bacteria, yeast, and mammals have generated a large pool of data about the genetic control and biochemistry of NER and have also uncovered many structural and mechanical details of the lesion processing events. The last decade of intensified research in plant DNA repair field has revealed that most of the key NER genes are present in both model and crop plants, and notably many repair factors are identified as being components of similarly structured protein complexes. Moreover, in plants many NER genes have been interconnected to HR and photorepair and such a dynamic interplay of different DNA repair mechanisms may contribute to the adaptability and plasticity of plant genomes (Dubest et al., 2004; Molinier et al., 2008).

The *AtCEN2* gene has been implicated in the early steps of plant GGR as part of the AtRAD4 (XPC) recognition complex. It was also shown to provide a link between NER and HR as an alternative mechanism for CPD repair in plants (Molinier et al., 2004; Liang et al., 2006). The role of the AtGTF2H2 protein in both transcription and NER has been inferred by its interaction with AtXPD helicase and further supported by its ability to act in the transcription and repair processes in yeast (Vonarx et al., 2006). In addition, AtGTF2H2, AtXPD, AtTFB1A, and AtTFB5 have been identified as being the *Arabidopsis* homologs to the human TFIIH factors p44, XPD, p62, and TTDA, respectively. All of them were shown to be components of the plant TFIIH complex and a structural model for its core assembly has been developed based on protein interaction data (Grice et al., 2007). Two *DDB1* homologs are present in the *Arabidopsis* genome (*DDB1A* and *DDB1B*), whereas in rice and tomato the *DDB1* is a single-copy gene. Genetic and molecular analyses have provided plenty of data about the involvement of CUL4-DDB1-DDB2 E3 ligase complex in plant GGR. The *CUL4, DDB1A*, and *DDB2* insertion mutants in *A. thaliana* are hypersensitive to UV-C radiation and deficient in the dark repair of UV lesions (Molinier et al., 2008). In accordance with this, *AtDDB1A* overexpression improved the immediate response to the damaging UV light and was also necessary for the UV-induced regulation of the other *DDB1B* and *DDB2* genes (Al Khateeb and Schroeder, 2009). The expression of the *OsDDB* gene was also shown to increase upon UV exposure but mainly in the meristematic rice tissues (Ishibashi et al., 2003). In addition to their involvement in NER, plant DDB factors have shown a broad range of protein interactions that appear critical for the normal development and physiology at different stages of the plant growth (Ganpudi and Schroeder, 2013). Mutations in *AtXPD/UVH6, DDB1A*, and *DDB2* were found to influence both plant UV and heat response, and also cause defects in the floral development (Ly et al., 2013). In tomato, DDB1 has been linked to organogenesis, photomorphogenic response and even to the nutritional quality of the fruit, thus assigning a direct role of DNA repair-related genes and processes to the value of crops (Azari et al., 2010; Liu et al., 2012).

In plants the preferential removal of CPD and/or other DNA lesions from actively transcribed genes by NER pathway has not been a subject of intensive research, although repair heterogeneity has been proposed to occur in maize (Stapleton et al., 1997). In *Arabidopsis*, however, it has been recently shown that, in contrast to the low-rate repair observed at the whole genome level in the dark, the housekeeping RPII gene has been efficiently restored in a period of 24 h after UV-B irradiation. The more intriguing observation was that such repair was due to the enhanced CPD removal from the transcribed strand of the gene, thus providing a direct evidence for active TCR in the plant genome (Fidantsef and Britt, 2012). Consistent with these results *AtCSA*-like genes have been implicated in the regulation of TCR and UV damage response as part of the CUL4-DDB1^CSAat1A and B^ complex in *A. thaliana* (Zhang et al., 2010a). The expression of the AtCSA-1 protein was found to be constitutively high in all plant tissues and therefore not upregulated by UV-B. However, its distribution in the nucleus showed a pattern of speckles in areas with high transcriptional activity, thus supporting involvement in TCR (Biedermann and Hellmann, 2010). Therefore, the selective operation of excision repair pathways at the level of actively transcribed genes seems really important for plants (i.e., more than previously appreciated) and it would be interesting to explore whether gene-specific repair might contribute to the UV tolerance in crops as well.

### Mismatch Repair

The main biological role of the MMR system is to correct errors such as mismatches or nucleotides accidentally inserted/deleted during replication. In addition, MMR participates in the correction of mispaired bases and loops in the recombination intermediates, rejection of excessive heteroduplexes, removal of exogenous DNA lesions such as psoralen-induced interstrand cross-links, oxidative DNA damage and UV photoproducts, as well as in nucleosome remodeling (Wu et al., 2005; Javaid et al., 2009; Lario et al., 2011; Honda et al., 2014). Hence, an efficient MMR helps the cell to increase the fidelity of DNA replication, to decrease the rate of mutations, to control the dynamics of short repetitive sequences, to maintain genome integrity, to conduct high-fidelity homologous and inhibit homeologous recombination, as well as to carry out proper meiosis (Spampinato et al., 2009). As an essential safeguard of genomic stability the MMR system is highly conserved in all living organisms, although some variations between the kingdoms were found to exist. The main prokaryotic MMR genes MutS and MutL have multiple homologs in the eukaryotes known as *MSH* and *MLH* gene families. Most of the eukaryotes contain at least six *MSH* genes, whereas the seventh, *MSH7*, is specific for plants (Culligan and Hays, 2000). MSH polypeptides combine in various heterodimeric complexes such as MSH2•MSH6 (MutSα), MSH2•MSH3 (MutSβ) or MSH4•MSH5, which are more or less specialized for certain DNA structures or DNA damage. They function in either replicative or recombination MMR, or in both. In general, the MSH heterodimers have lesion recognition and DNA binding activities, whereas the actual repair reactions initiate upon recruitment of the MLH heterodimer (Culligan et al., 2000). The endonuclease activity of MutLα (MLH1-PMS2) is particularly important for the strand-specific correctness of mismatch removal. Other proteins implicated in the downstream stages of MMR are Exo1, PCNA, RPA, RFC, and DNA polymerase δ (Modrich, 2006). In replicative MMR the main difference between bacteria and eukaryotes is in the mechanism used to differentiate between the inaccurately inserted and the normal nucleotide within the mismatch. In *Escherichia coli* the newly synthesized strand is easily distinguished because it initially lacks the methylation of specific DNA sequences. In eukaryotes the exact recognition mechanisms are not yet well understood, but it is now evident that they involve a tight interplay between replication and MMR (Jiricny, 2013). Indeed, data in yeast suggests that in the course of replication the MMR factors are temporarily coupled to the replication machinery which allows them to recognize repair signals elicited from the daughter strand shortly after replication (Hombauer et al., 2011). Up to now, eukaryotic MMR reactions have been fully reconstituted *in vitro* with both human and yeast MMR enzymes (Bowen et al., 2013), however, such studies are still to be performed in plants.

Similar to other eukaryotes, plants rely on the proper function of MMR factors during post-replicative and recombination MMR to preserve their genomic stability. In *Arabidopsis*, *MSH2* deficiency inhibits homologous but increases homeologous recombination (Li et al., 2006) and enhances the microsatellite instability particularly in germline cells (Leonard et al., 2003), whereas *MSH7* controls meiotic recombination *(*Lario et al., 2015). In cereals, the loss of *MSH7* gene function impairs meiotic recombination and reduces plant fertility (Lloyd et al., 2007). Inactivation of AtMLHs leads to disruption of all MMR-dependent processes such as meiosis, mitotic recombination, and damage removal (Dion et al., 2007). Plant MMR is not limited to the nucleus; the nuclear-encoded MSH1 protein also localizes in the extranuclear genomes of both dicots and monocots, where it influences different chloroplast and mitochondrial functions (Xu et al., 2012b). AtMSH polypeptides associate *in vitro* to form the known MSH2•MSH6 (AtMutSα) and MSH2•MSH3 (AtMutSβ) heterodimers, which exhibit binding efficiencies and mismatch substrate specificities similar to the other eukaryotic homologs, whereas the AtMutSγ (MSH2•MSH7) protein complex shows specific substrate binding affinity which suggests more specialized functions (Wu et al., 2003). It was recently demonstrated that overexpression of plant MutS and MutL proteins in yeast cells significantly disturbs MMR function and consequently destabilizes the yeast genome. A specific role of AtMutSγ in the recognition of mismatches generated at the sites of spontaneous or stress-induced DNA lesions has also been proposed (Galles and Spampinato, 2013; Gómez and Spampinato, 2013). Indeed, data have shown that plants deficient in *AtMSH7*, *AtMSH2*, or *AtMSH6* genes when exposed to high UV-B levels, have higher CPD content, reduced CPD repair kinetics, as well as impaired cell cycle progression compared to the MMR proficient ones. In addition, wild type *Arabidopsis* and maize respond to UV-B irradiation by enhancing the expression levels of *MSH2* and *MSH6* genes, consistent with the role of the MMR system in the UV defense strategy of both model and crop plants (Lario et al., 2011, 2015).

There is an increasing demand to expand the current knowledge on the MMR system from model to crop species as such studies may have direct impact on the modern agriculture. Mutation-based breeding and introgressive hybridization have long been powerful tools in crop improvement and both these processes have been linked to the functionality of MMR in plants (Tam et al., 2009). It was suggested that inactivation of MMR may help induce mutagenesis with lower associated toxicity, as lower doses of mutagens would be sufficient to obtain desired traits in an MMR deficient background (Hoffman et al., 2004). MMR deficiency alone was sufficient to significantly increase the mutation rate in *Arabidopsis* without any mutagenic treatment, showing a mutation spectrum different from that achieved after EMS treatment. Therefore, in *Arabidopsis* an approach based on the reversible inhibition of MMR genes was successfully utilized to obtain plants with selectable phenotypes (Hoffman et al., 2004; Chao et al., 2005). The efforts to manipulate the MMR system are not confined to the *Arabidopsis* genome but have recently been employed in some crop species such as rice, tobacco, and tomato (Tam et al., 2011; Xu et al., 2012a; Van Marcke and Angenon, 2013). Various strategies have been applied such as RNAi-induced silencing and/or dominant negative suppression of MMR repair genes and all these techniques seem promising for the future agriculture to overcome the barriers between distantly related species or enhance mutation variability.

### Recombinational Repair and Non-homologous End Joining

#### Detection and Signaling of DSB Occurrence

The lack of plant genome stability may cause abnormalities in plant development and, in the case of crop plants, yield reduction. DSBs pose a major threat to genome stability because if not repaired before cell division they may lead to loss of substantial genetic material (Puchta, 2005; Feuerhahn and Egly, 2008; Charbonnel et al., 2010). DSBs have also been shown to cause programmed cell death in plants (Roy, 2014). In all eukaryotes, in the course of evolution, a complex system has been established, which includes DNA damage recognition and response pathway (DDR) and cell cycle checkpoint components. The DNA damage response mechanism and components of the cell cycle regulation coordinate DNA repair in the context of cell cycle phase. In order to initiate DSB repair, and to signal the occurrence of the DNA damage and recruit the repair proteins within the affected region of a nucleus, structural, and chemical modification of nucleosomes at the damaged site takes place, as shown in eukaryotes (van Attikum and Gasser, 2009). The primary signal transducers of DNA breakage are two phosphatidylinositol 3 kinase-like (PI3K) protein kinases: ATM and ATR. Both kinases initiate a phosphorylation-mediated signal transduction cascade that leads to cell-cycle arrest and repair of DSBs. The ATM and ATR kinases phosphorylate the histone variant H2AX in a large chromatin domain around the damage. This phosphorylation induces the accumulation of other damage-response factors and produces cytologically detectable foci (Amiard et al., 2011).

*Arabidopsis atm* mutants are sensitive to DSB-inducing factors, whereas the *atr* mutant plants are sensitive to replication stress (Garcia et al., 2003; Culligan et al., 2004, 2006). A role of these proteins in the DDR signaling in plants was validated by the fact that H2AX phosphorylation in response to irradiation-induced DSBs is dependent on ATM (Friesner et al., 2005). In plants a signal of the presence of a DSB is transduced by the ATM kinase, whereas the signal communicating the presence of stretches of a single-strand DNA is transduced by the ATR kinase (Yoshiyama et al., 2013). Eukaryotic genomes encode histone variants, which differ from canonical histones in amino-acid sequence and are encoded by single-copy genes. These histone variants are associated with various genomic regions and play major roles in regulation of transcription, chromatin condensation, and in DNA damage response and DNA repair (Law and Cheung, 2013). As a consequence of the presence of a DSB, histone H2AX is phosphorylated at the C-terminal part of the protein (histone variant γH2AX) forming the γH2AX foci (van Attikum and Gasser, 2009). It was reported that the γH2AX foci constitute sites of recruitment of various DNA repair proteins (Paull et al., 2000). There is a correlation between the number of γH2AX foci and the number of DSBs, as well as between the rate of removal of the phosphorylated histones H2AX and efficiency of DSB repair. In fact, measurement of γH2AX foci formation is considered several orders of magnitude more sensitive than other methods of DSB detection (Bonner, 2003; Löbrich et al., 2010). This approach to measurement of DSB repair was successfully applied in plant genomes (Friesner et al., 2005; Charbonnel et al., 2010), including crop species with relatively large genomes, such as barley (Stolarek et al., 2015a,b). Recently, in *Arabidopsis*, a land-plant-specific transcription factor SOG1 has been identified. It functions as a central regulator in DNA damage response and performs functions analogous to mammalian p53, being involved in the majority of plant response to DNA damage, such as transcriptional response, activation of cell cycle checkpoint and programmed death (Yoshiyama et al., 2014).

#### Mechanisms of DSB Repair

**FIGURE 4:**
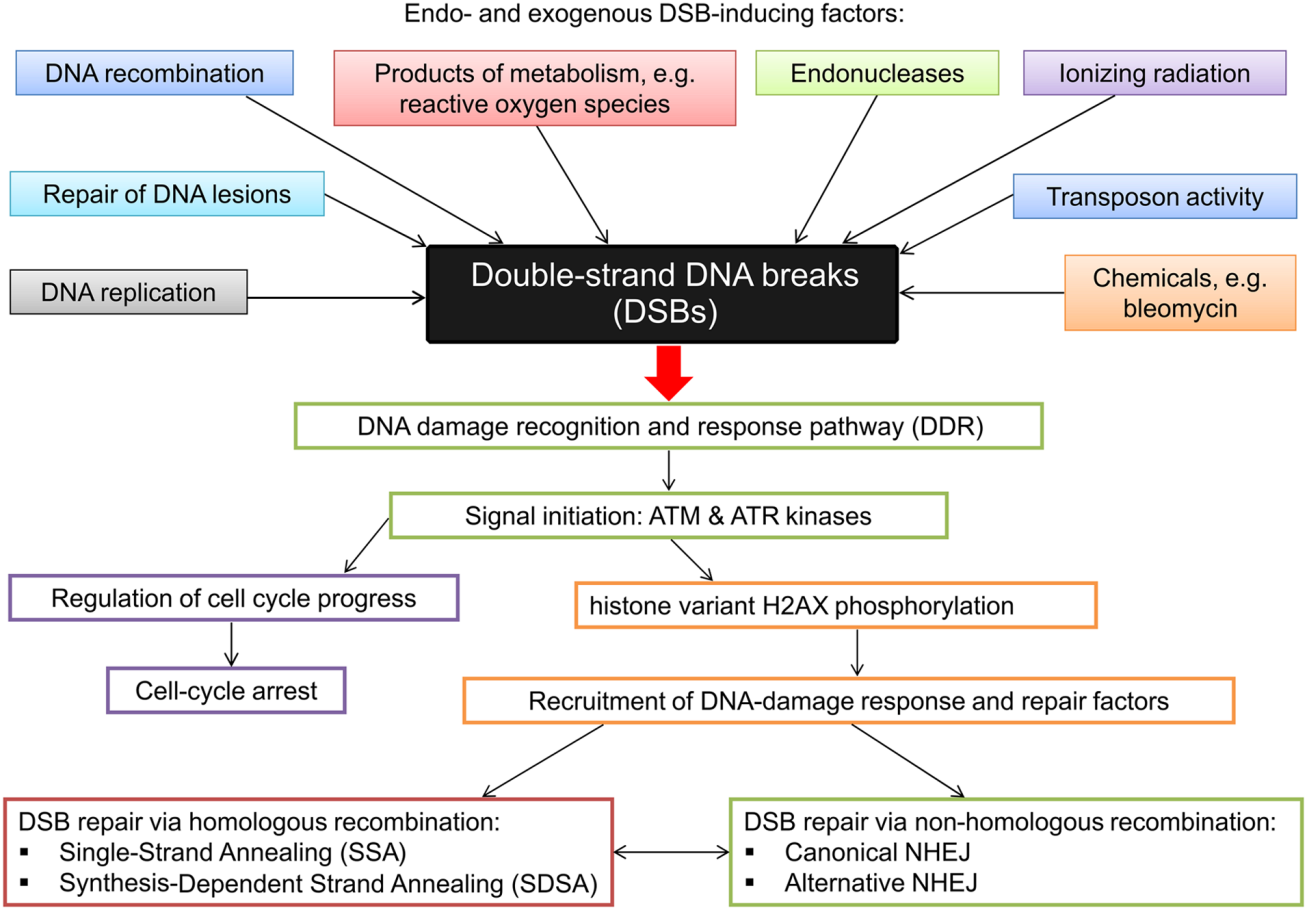
**Diagrammatic representation of the DSB inducing factors and the various damage recognition and repair pathways**.

Upon DSB detection two different repair mechanisms may be initiated to repair the lesion – HR and non-homologous end joining. In DSB repair via HR the broken DNA ends are repaired based on the regions of sequence homology, whereas in NHEJ the sequence information does not play a significant role in re-joining of DSBs (Puchta, 2005). The latter mechanism does not require homologous template sequence and is therefore error-prone (Charbonnel et al., 2010). HR can also be error prone if not restricted to the identical locus on a sister chromatid. The principle mechanisms and basic factors in HR-mediated DSB repair and NHEJ are conserved in eukaryotes (Puchta and Fauser, 2014). Studies aimed at characterization of DNA repair processes in plants have been conducted mainly in *Arabidopsis*. Our previous studies indicated that, based on high conservation level of these processes across various evolutionary groups of organisms, gene, and protein sequences related with DNA repair may be deployed as queries for browsing databases to identify homologous sequences in other species, including crop plants, in which these mechanisms are poorly understood (Gruszka et al., 2012). Application of various approaches in plant systems and sequencing of re-joined sites allowed identification of specific features of DSB repair in plants: end joining is usually associated with various sequence rearrangements (insertions, deletions) ranging up to 1 kb, and rejoining frequently takes place at sites of short repeats. Moreover, the sequences inserted at the re-joined site may be derived from other locations in a genome – consequently NHEJ in plants appears more error-prone than in other organisms (Gorbunova and Levy, 1999; Puchta, 2005). Analysis of DSB joining in two plant species varying significantly in their genome size – *Arabidopsis* and tobacco - indicated that the pattern of sequence alterations at the junction sites varies between the plant species and that large deletions at the junction sites are more frequent in smaller genome of *Arabidopsis* (Kirik et al., 2000; Lloyd et al., 2012). This indicates that error-prone NHEJ may contribute significantly to the evolution of plant genome size (Puchta, 2005). It is now known that high-fidelity DSB repair via HR is less frequent in plant genomes than NHEJ (Puchta and Fauser, 2014). On the other hand, in plant genomes, especially the large ones, with high content of repeated sequences, recombination between two non-allelic sequences may lead to crossovers and genome rearrangements and in this situation NHEJ proves safer than HR. The generally used classification of HR-mediated repair versus NHEJ may be oversimplified because, especially in plants, both mechanisms act together, and many rearrangements observed in plant genomes may be explained by a combination of these pathways. Although DNA repair in lower organisms usually is more accurate, accuracy of DNA repair cannot be simply correlated with genome size or amount of repetitive DNA (Gorbunova and Levy, 1999). Mechanisms of DSB emergence and induction as well as various damage recognition and repair pathways are presented in **Figure 4**.

##### Non-homologous end joining

Upon detection of a DSB and recruitment of the above-mentioned signaling components several repair pathways may be activated. According to the current classification the NHEJ pathway is divided into several sub-pathways, including canonical or cNHEJ and the recently discovered aNHEJ sub-pathway (Mladenov and Iliakis, 2011).

The cNHEJ mechanism is highly conserved among both pro- and eukaryotic organisms. It is involved in the repair of DSBs that are produced by both physical and chemical factors, but also as a result of V(D)J recombination in animals, and T-DNA integration into a genome during transformation (Charbonnel et al., 2010; Lieber, 2010; Charbonnel et al., 2011; Boboila et al., 2012; Park et al., 2015). The process is initiated by damage recognition mediated by the heterodimer Ku70/Ku80 forming a ring structure, which binds both DNA ends, brings them together and prevents their degradation (Walker et al., 2001; Mannuss et al., 2012). Therefore, cNHEJ often causes a minor change in genetic information (Puchta and Fauser, 2014). The step mediated by the Ku70/Ku80 heterodimer is followed by the recruitment of DNA-PKcs kinase, which promotes or restrains the access to damaged DNA ends. However, the DNA-PKcs kinase has not been identified in plants (Nishizawa-Yokoi et al., 2012). At the site of the lesion the DNA ends are processed, as required, by several proteins to restore the correct phospho- and hydroxyl-groups at both the 5′ and 3′ DNA ends, respectively. The cNHEJ pathway is terminated by DNA ligation, which is mediated by various proteins, such as the LigIV/XRCC4, XLF, PARP3 [Poly(ADP-ribose) polymerase 3] and the APLF protein (Neal and Meek, 2011; Rulten et al., 2011).

Key players participating in cNHEJ, such as Ku70, Ku80, XRCC4, and ligase IV have been identified in *Arabidopsis*. Mutations in the *Ku70*, *Ku80*, and *LigIV* genes lead to increased sensitivity to DSB-inducing factors (West et al., 2000; Tamura et al., 2002; West et al., 2002). Inactivation of the Ku80 protein in *Arabidopsis* considerably reduces the efficiency of cNHEJ. However, surprisingly *Arabidopsis ku70*, *ku80*, and *ligIV* mutants do not show growth defects or increased inviability (Bleuyard et al., 2006). *Arabidopsis* Ku70 and Ku80 proteins were shown to bind double-stranded DNA (non-telomeric and telomeric sequences), but not single-stranded DNA. Both proteins possess ATPase and ATP-dependent DNA helicase activities. Expression of these genes is increased in response to DSB-inducing factors such as BLM and MMS (Tamura et al., 2002). Orthologs of XLF have not been identified in plants. Three genes encoding proteins similar to Artemis were identified in the *Arabidopsis* genome, but their role in cNHEJ was not validated (Charbonnel et al., 2010). The key mediator of the cNHEJ process – the Ku70/Ku80 heterodimer is also suggested to function as a repressor of HR within telomeric sequences, which may lead to the shortening of telomeres. In *Arabidopsis* the involvement of the Ku80 protein in telomere length regulation was confirmed and the *atku80* mutant has longer telomeres than wild type (Gallego et al., 2003; Fattah et al., 2010). The function of the Ku70/Ku80 heterodimer has been validated in rice (Nishizawa-Yokoi et al., 2012) and wheat (Gu et al., 2014). Recently, barley mutants carrying mutations in the *Ku80* gene were identified. These mutants accumulated BLM-induced DSBs to a much greater extent than the parent cultivar. The study also demonstrated a significant role of the *HvKu80* gene in the regulation of telomere length in barley (Stolarek et al., 2015b).

In the less well-characterized aNHEJ process, 3′ resection of the broken DNA ends occurs. Annealing of the two single strands leads to junction formation at the sites where few complementary nucleotides are present. The end flaps are usually trimmed, re-joining occurs and the microhomologies get exposed at the junction site. As a result of DSBs resection, the aNHEJ process usually leads to deletions at the junction site (Puchta and Fauser, 2014). It was reported that the PARP1 and XRCC1 proteins are involved in this process and are conserved both in mammals and plants (Charbonnel et al., 2011; Jia et al., 2013). Very often genetic information at the junction site is lost, therefore the aNHEJ process is considered as highly mutagenic way of DSB repair (Puchta and Fauser, 2014). It was also demonstrated that both NHEJ sub-pathways compete for DSB repair. In the *Arabidopsis* mutant *ku80* occurrence of the error-prone re-joining (aNHEJ) was increased by 2.6-fold and higher frequency of end degradation was observed (Osakabe et al., 2010). It was reported that an additional NHEJ pathway may be responsible for repair of some DSBs in the absence of cNHEJ and aNHEJ (Charbonnel et al., 2011). The function of the *PARP* genes in *Arabidopsis* is still far from being fully elucidated, however, it was recently suggested based on high expression level of this gene in seeds, that it may play a crucial role in DNA damage response at this developmental stage. Upon seed imbibition ROS are produced, which may result in DNA damage. Under these conditions DNA repair is critical to maintain the genome integrity of developing embryo (Balestrazzi et al., 2011; Boltz et al., 2014). Based on the information from model species – *Arabidopsis* and rice, recently the barley *PARP3* homolog has been identified. Expression of the *HvPARP3* gene is enhanced by the DSB-inducing chemical BLM. This result indicates that *HvPARP3* functions as a component of DNA damage-response system. Mutational analysis of the gene led to the identification of a series of alleles. Mutation of the amino-acid residue located in a highly conserved domain evoked a significant increase in the number of DSBs produced after the mutagenic treatment, which was measured with various approaches (Stolarek et al., 2015a).

##### Homologous recombination

In somatic cells of plants HR is a minor DSB repair pathway functioning mainly during the S and G_2_ phases of the cell cycle. The two most prominent HR-dependent pathways of DSB repair in plant somatic cells are SSA and SDSA (Puchta, 2005). The most common intermediate step of the DSB repair process is resection of DNA ends and exposure of single-stranded 3′ overhangs. This was proposed as the first step for the majority of DSB repair pathways in plants and seems to be mediated by the MRN complex. Plant homologs of MRE11, RAD50, and NBS1, which form the MRN complex, have been identified (Hartung and Puchta, 1999; Gallego and White, 2001; Akutsu et al., 2007). In *Arabidopsis* the functions of two components of this complex – AtMre11 and AtRAD50 were validated and co-immunoprecipitation assays indicated that these two proteins form a complex (Gallego et al., 2001; Bundock and Hooykaas, 2002; Daoudal-Cotterell et al., 2002). The MRN complex is also required for γH2AX phosphorylation by the ATM and ATR kinases in response to DNA damage (Amiard et al., 2010). It is suggested that in *Arabidopsis* the MRN (Mre11-Rad50-Nbs1) complex may also be recruited to DSBs by the Ku70/Ku80 heterodimer during the NHEJ pathway (Tamura et al., 2002). Characterization of the *atmre11* and *atrad50* mutants indicated conservation in the functions in recombination and DSB repair of the *Arabidopsis* MRN complex (Bleuyard et al., 2006). Inactivation of the *AtMre11* and *AtRAD50* genes conferred a phenotype of hypersensitivity to DSB-inducing factors, but the mutant plants remained viable (Gallego et al., 2001; Bundock and Hooykaas, 2002). However, sterility observed in these mutants suggests conservation of the role of the MRN complex in HR during meiosis (Gallego et al., 2001; Puizina et al., 2004). Cytological analyses of meiotic cells indicated that the sterility is caused by DNA fragmentation during meiotic prophase (Puizina et al., 2004). Another factor important for the initiation of HR is the RPA, which is a eukaryotic heterotrimeric protein complex that binds single-stranded DNA. In plants, multiple genes encode the three RPA subunits (RPA1, RPA2, and RPA3; Ishibashi et al., 2006; Shultz et al., 2007; Atwood et al., 2014; Eschbach and Kobbe, 2014). The RPA complex plays essential roles in various DNA metabolic pathways, including DNA replication, meiotic recombination, and repair. This complex participates also in the activation of the cellular response to DNA damage (Aklilu et al., 2014). Moreover, in *Arabidopsis*, one of the subunits (RPA1) takes part in the NHEJ process and negatively regulates the telomere length (Takashi et al., 2009).

According to the SSA model the two single-stranded 3′overhangs may anneal at the site of microhomology (Gorbunova and Levy, 1999). In *Arabidopsis* and other model organisms this process is mediated by the Rad52 protein (Samach et al., 2011). The SSA process may only occur when the DSB is located between two homologous/complementary sequences. If the resulting DNA molecule contains non-complementary overhangs they are removed, and the remaining single-stranded gaps are filled by DNA polymerase and the repair is completed by ligation by DNA ligase I. The SSA pathway is a non-conservative mechanism, as it usually leads to deletions of sequences which were originally located between repeated sequences of microhomology (Puchta and Fauser, 2014). The SSA mechanism seems to be of great importance for molecular evolution of genomic regions with tandem duplications. In these regions up to one-third of DSBs is repaired with the use of the SSA pathway (Siebert and Puchta, 2002). The SSA mechanism leading to deletion of sequences located between direct repeats is particularly efficient. It may explain the abundance of single LTRs as remnants of retrotransposons in cereal genomes (Puchta, 2005).

The other DSB repair model – SDSA – is based on invasion of a homologous, double-stranded template molecule with a single-stranded 3′ overhang, which is then extended during DNA synthesis. The single-stranded 3′ overhang invasion into the template molecule is most likely mediated by the RAD51 protein and its paralogs (Bleuyard et al., 2005; Osakabe et al., 2005). In the *Arabidopsis* genome *AtRAD51* is a single-copy gene, and its transcription is increased after gamma-irradiation (Doutriaux et al., 1998; Bleuyard et al., 2006). It was reported that the *atrad51* mutants are viable, even though the mutants showed a severe sterility phenotype due to defects in synaptonemal complex assembly and chromosomal instability during meiosis (Li et al., 2004). Most probably the formation of the RAD51/ssDNA nucleofilament is also mediated by *Arabidopsis* orthologs of the BRCA2 protein, which interacts directly with AtRAD51. Similar to the *atrad51* mutants, *Arabidopsis* RNAi lines in which the *AtBRCA2* gene was knocked-down showed partial sterility caused by chromosomal instability during meiosis (Siaud et al., 2004). Contrary to the SSA pathway, the SDSA mechanism is conservative, as homology from the donor sequence (template DNA molecule) is copied into the DSB without any loss of DNA sequence (Puchta and Fauser, 2014). The SDSA pathway was reported to be five to ten times less efficient in DSB repair than the SSA mechanism under comparable conditions (Orel et al., 2003). The mechanism of SDSA explains the frequent events of gene conversion or insertion of filler DNA at the re-joining site. During this process the single 3′ end invades a homologous double-stranded, template DNA molecule, forming a D-loop structure (Puchta and Fauser, 2014). It is now known that both the 3′ single-stranded overhangs may act independently in search for a template molecule, which is to be invaded. Therefore, in the SDSA mechanism both homologous and non-homologous templates may be invaded (Gorbunova and Levy, 1999). The SDSA mechanism is considered a conservative mode of DSB repair and usually results in gene conversions, however, without Holliday junction formation and crossover events. This mechanism seems to play a significant role in molecular evolution of tandemly arranged gene families (Puchta, 2005). During the DNA synthesis interaction between the template and the extended strand is weak, and therefore abortion of synthesis and template switch may occur. Multiple template switches result in complex ‘patchwork’ DNA inserts at the re-joining site (Gorbunova and Levy, 1999). The SDSA mechanism may constitute the most versatile model of genomic DSB repair in somatic plants cells, as it is based on one-sided initiation and may combine both HR and NHEJ events. If the 3′ end of the invading strand is elongated up to the homology with the second 3′ end of the DSB (available due to resection) both single strands may anneal (microhomology-based second end capture). However, if the 3′ end of the invading, elongated strand is not complementary to the other 3′ end of the DSB the break is ultimately repaired via NHEJ. In contrast to the SSA mechanism, no sequence is lost as a result of the SDSA process, however, the information content may be altered (Puchta, 2005; Puchta and Fauser, 2014). Normally, sequences in close proximity on the same chromosome or sister chromatid are used as templates for SDSA, and ectopic or allelic homologies are rarely used in DSB repair (Gisler et al., 2002).

As suggested by the different mechanisms of the SSA and SDSA pathways, groups of DNA repair-related proteins mediating both processes are quite different. The strand exchange results in formation of an intermediate of the SDSA process, therefore the RecA homologs RAD51 and XRCC3 were identified as mediators of this process in *Arabidopsis*. Additionally, AtRAD54 being an ATPase and belonging to the SWI2/SNF2 family of molecular remodelers of DNA structure, is also essential for the SDSA process (Roth et al., 2012). The AtRECQ4A and AtFANCM DNA helicases, and nucleases, such as AtMUS81, play a role in SDSA, but only a minor in the SSA pathway (Mannuss et al., 2010). In the case of the SSA pathway the RAD1/RAD10 heterodimer, which functions as a structure-specific flap-like endonuclease, is involved in trimming of the complementary strands before ligation (Dubest et al., 2002). No other factors, which would be essential for the SSA mechanisms have been identified yet (Puchta and Fauser, 2014).

The classical HR model of DSB repair involves formation of a Holliday junction, and resolution of this structure resulting in gene conversion and crossover. In this mechanism both strands of the template molecule are simultaneously used during extension of both single-stranded 3′ overhangs (Gorbunova and Levy, 1999). However, it should be noted, that homology to only one end of the DSB is sufficient for initiation of HR, and DSB repair in plant cells may be initiated by a one-sided interaction event (Puchta, 2005). It is now suggested that both DSB repair via HR and Holliday junction formation and SDSA mechanism may be utilized in plant cells. However, depending on the context – Holliday junctions are formed predominantly during meiosis when fidelity of repair is assured by pairing of homologous chromosomes (Keeney, 2001), while the SDSA mechanism functions mainly in somatic cells (Puchta, 2005).

Both the SSA and SDSA pathways may be part of homologous and non-HR. However, comparison of efficiencies of both pathways indicated that SSA mechanism is about five times more efficient than the SDSA mechanism. Therefore, the SSA mechanism may be the most prominent homology-based way of DSB repair in higher eukaryotes, in general. However, an accumulating body of evidence derived from various approaches and experiments indicates the most efficient way of DSB repair in plant somatic cells is NHEJ. Nevertheless, if homologous sequences are available during the repair process, in one-third of the cases DSBs are repaired via the SSA pathway, and about six percent of the DSB repair events proceed via the SDSA model (Puchta, 2005).

## Site-Specific DSB Induction as a Tool for Targeted Mutagenesis

Rare cutting endonucleases have been applied as tools for induction of DSBs and the analysis of many aspects of DSB repair in plant genomes (Mannuss et al., 2010; Roth et al., 2012; Wei et al., 2012). Combining knowledge about mechanisms of DSB repair, their consequences and application of sequence-specific endonucleases led to development of site-specific DSB induction as a tool for targeted mutagenesis. Alterations in a genome sequence are introduced by DSB formation, which induces natural repair mechanism. Synthetic nucleases may be applied to induce DSBs which lead to mutations through erroneous NHEJ-mediated DSB repair. Site-specific DSB induction may also be a starting point for transgene integration into a genome via NHEJ or HR pathway (Voytas, 2013; Puchta and Fauser, 2014). It was shown that ZFNs may be used for sequence-specific mutation induction in the *Arabidopsis* genome via NHEJ (Lloyd et al., 2005) and as an efficient approach for knockout of *Arabidopsis* genes via the same DSB repair mechanism (Osakabe et al., 2010; Zhang et al., 2010b). It was also reported that ZFNs may be applied for gene-targeted mutagenesis in tobacco and maize by DSB-induced HR (Shukla et al., 2009; Townsend et al., 2009). ZFNs became a very efficient tool for genome editing in plants and the list of plant genomes that may be modified in the site-specific DSB-dependent manner is constantly growing (Weinthal et al., 2010; Puchta and Fauser, 2014).

However, new alternatives emerged recently. TALENs are one of these very effective approaches to genome engineering (de Lange et al., 2014). Recent studies have shown a huge potential application of TALENs for targeted plant genome mutagenesis (Christian et al., 2010; Mahfouz et al., 2011; Shan et al., 2013a; Wendt et al., 2013). An example of a trait obtained through TALEN-based targeted mutagenesis is a soybean variety that produces oil with elevated levels of the monounsaturated fat – oleic acid. The TALEN-based approach was deployed to mutate the *FAD2-1A* and *FAD2-1B* genes encoding fatty acid desaturases (Haun et al., 2014). Another system – CRISPR/Cas and associated endonuclease is an alternative tool to induce sequence-specific DSBs in model and crop plant genomes (Feng et al., 2013; Jiang et al., 2013; Li et al., 2013; Mao et al., 2013; Miao et al., 2013; Nekrasov et al., 2013; Shan et al., 2013b; Xie and Yang, 2013). In *Arabidopsis* protoplasts the frequency of NHEJ-based targeted mutagenesis reached 5.6%, whereas in *Nicotiana benthamiana* the frequency was up to 38.5% (Li et al., 2013). Efficient NHEJ-mediated targeted mutagenesis was also observed in rice protoplasts, however, with varied frequencies (14.5–38%), and in wheat protoplasts, at a frequency of 28.5% (Shan et al., 2013b). It was demonstrated in several studies that DSB induction by Cas endonucleases is useful for targeted mutagenesis, but also for HR-related techniques of plant genome engineering (Feng et al., 2013; Mao et al., 2013; Miao et al., 2013). Experiments aimed at DSB-induced genetic engineering are currently being developed for a number of plant genomes, and the CRISPR/Cas system has proved to be efficient in a number of model and crop plants; however, it should be kept in mind that efficient transformation and regeneration of transgenic material in some cereal crop species, like barley is still a challenge (Puchta and Fauser, 2014). Mechanisms of DNA repair have been studied for many years in basic research, and currently our understanding of these processes, and DSB repair in particular, becomes a crucial element of development of advanced tools for precise genome modification procedures and targeted mutagenesis.

## Small RNAs in Plant DNA Damage and Repair

RNA interference is a cellular mechanism for control of gene transcription via small RNAs (siRNAs, miRNAs, ta-siRNAs, etc.) which promote the degradation of their target mRNAs thus leading to gene silencing. Small RNAs are an excellent tool for suppression of gene expression in order to study the genetic control of various cellular pathways. More importantly, they have an enormous practical application in both human health and agricultural practice. Regarding the latter, in plants, small RNAs offer the possibility to manipulate and engineer plant genome and have been widely utilized to obtain desired traits in crop plants (Saurabh et al., 2014).

Over the last decade, RNAi-based technology has become a major breakthrough in DNA repair research. Its application allowed elucidation of the particular role the individual proteins and protein complexes play in different aspects of DNA damage control and repair. In mammalian cells, such studies have become a routine practice in many laboratories. In plants, experimentally generated transgenic RNAi lines have been extremely useful to reveal the interplay between HR and NER pathways in *Arabidopsis* (Molinier et al., 2004), to examine the link between chromatin modifications and UV-B damage response in maize and *Arabidopsis* (Casati et al., 2008; Fina and Casati, 2015), to identify a novel Lig1-dependent DSB repair pathway in *Arabidopsis* (Waterworth et al., 2009), etc. Nevertheless, the employment of RNAi in the studies on DNA repair in crop species remains to be expanded in the future.

In addition to the key role the different types of naturally occurring small RNAs play in various molecular processes in plants, such as stress defense, epigenetic control, and transposon suppression, it was recently shown that they might be involved, directly or indirectly, in DNA damage response and DNA recombination and repair. A computational approach utilizing microarray gene expression data from *Arabidopsis* identified a number of miRNA genes, which are upregulated in plants exposed to UV-B radiation, and determined that their targets code for different transcription factors or belong to the auxin signaling pathways (Zhou et al., 2007). It was shown that *Arabidopsis* mutants, deficient in the DCL enzymes or RNA dependent RPs, display differential response to MMS varying from higher sensitivity to higher tolerance compared to the wild type. In addition, *dcl2* and *dcl3* mutants have decreased ability to repair UV-C generated DNA lesions, suggesting a link between siRNA biogenesis and DNA repair (Yao et al., 2010). It was also shown that the DCL enzymes are involved in DSB repair in both *Arabidopsis* and human cells, possibly via production of small RNAs from sequences surrounding the DSB. It was proposed that these diRNAs mediate DSB repair and their recruitment occurs through the AGO2 protein, which is a central factor of the RISC complex (Wei et al., 2012). These findings were extended in mammalian cells and revealed that diRNAs and AGO2 are essential for the initiation of HR, where they facilitate RAD51 accumulation onto DNA (Gao et al., 2014). On the other hand, further experiments in *Arabidopsis* showed that *ago* mutant plants were not impaired in the meiotic recombination, however, the sensitivity of *ago2* as well as of *ago9* mutants to gamma radiation and mitomycin C was confirmed (Oliver et al., 2014). Generally, it might be expected that the new developments in RNAi-based technologies, combined with the knowledge on the mechanisms by which plants exploit small RNAs to activate and control their DNA damage tolerance and repair mechanisms under adverse conditions, would advance significantly the molecular plant breeding methods aimed at improving crop quality and production.

## Implication of DNA Damage and Repair In Biotic Stress Tolerance in Plants

In field conditions plants are not exposed to a single type of stress, but rather have to accommodate quickly to a combination of abiotic and biotic stress factors. In this respect, the recent research points to complex interconnections between the formation of DNA damage, activation of DNA repair pathways and plant defense response to pathogen attack. DSBs were detected in the *Arabidopsis* genome upon various types of pathogenic infection. The formation of these infection-induced DSBs was independent of the ROS production as a part of the host response. Moreover, it was found that the activation of pathogenic defense pathways also contributed to the maintenance of genomic integrity (Song and Bent, 2014). A number of studies have shown that HR was strongly enhanced in plants exposed to biotic stress and, more intriguingly, HR frequency was kept higher in their progeny as well (Molinier et al., 2006), accompanied with locus specific changes in DNA methylation pattern (Boyko and Kovalchuk, 2011). In addition, repair proteins engaged in HR have been implicated in the transcriptional control of genes involved in plant immune response (Song et al., 2011). On the other hand, it was demonstrated that the presence of UV lesions enhanced plant tolerance to pathogenic infection, but an inverse correlation was found between plant’s ability to repair those lesions and its pathogenic resistance (Kunz et al., 2008). It appears that the occurrence of certain levels of DNA lesions in the plant genome may be beneficial for plants to promote tolerance to pathogenic infection by keeping their defense pathways active. Overall, accumulating evidence shows that plants depend on DNA repair not only to overcome both abiotic and biotic stresses, but also to ensure better stress adaptation in further generations. Thus, in the long-term, revealing the mechanisms which govern such interactions may help to improve crop plants to better adjust to the dynamic environment.

## DNA Repair in the Context Of Mutation Induction and Crop Improvement

Although DNA damage is often considered regarding its mutagenic effect, the persistence of damaged bases also has a significant growth-inhibitory influence. Because the genetic variation created in part through mutation and recombination are prerequisites of both natural and artificial selection, the understanding of mechanisms of genetic change is relevant for both – our knowledge of evolution and for genetic manipulation of crop plants. Techniques of mutagenesis are a very efficient tool to develop necessary germplasm collections in model and crop species, facilitating discovery of desired loci and alleles. Identification of genes participating in these processes may shed light on molecular mechanisms of DNA repair. Induction of mutations within genes involved in DNA repair or lesion-tolerance pathway may alter the efficiency of these processes and, as a consequence, render the mutagenesis more effective.

Recently performed studies conducted with the use of bioinformatics tools enabled outlining the list of genes participating in various pathways of DNA damage repair in *A. thaliana* (Gruszka et al., 2012). However, information regarding mechanisms of DNA damage repair in crop plants is very limited. Understanding of the molecular basis of DNA repair and genome maintenance may allow more directed and fine-tuned mutation-induction techniques. It is important to take into account the global economic impact of the development of induced mutant-derived crop species (Ahloowalia et al., 2004).

During the last seven decades, worldwide more than 3200 varieties have been released, that were derived as direct mutants or from their progenies (Pathirana, 2011; IAEA Mutant Database^1^). Direct development of mutant varieties has been achieved mainly through application of radiation-based mutagenesis, mostly gamma and X-rays. Application of IR allowed development of important traits in crop species: resistance to bacterial leaf blight and blast in rice (Xiao et al., 2008), resistance to yellow mosaic virus in barley (Tanaka et al., 2010), resistance to potato virus Y in tobacco (Hamada et al., 1999), and resistance to black sigatoka in banana (Reyes-Borja et al., 2007). In a review by Pathirana (2011) a list of disease-resistant mutants developed directly through gamma irradiation in various crop species is presented. Mutant-derived varieties have been released in 175 plant species, including many important crops, such as rice, wheat, barley, cotton, rapeseed, sunflower, sesame, grapefruit, and banana. Many of these varieties have significant economic importance. For example, according to a report of the Japanese Science and Technology Agency, eighteen mutant-derived varieties of rice contributed US$ 937 million annually to the Japanese agriculture (Ahloowalia et al., 2004; Pathirana, 2011). The gamma ray-induced rice cultivar ‘Zhefu 802’ with a shorter growing season, cold tolerance and high yield potential under low-input conditions was the most widely cultivated variety in China for almost a decade. During this period its overall planted area reached 10.6 million ha (Kharkwal and Shu, 2009). The development of mutant-derived varieties and research on mutation techniques and molecular mechanisms of DNA damage and repair have long been supported by the Food and Agriculture Organization (FAO) and International Atomic Energy Agency (IAEA). This effort is particularly important in light of the rapidly growing world population and the need to improve and increase food production in the next few decades. However, in many countries, especially in Europe, issues concerning introduction of genetically modified crop varieties persist. Mutation-derived varieties meet criteria of non-GMO plants and may therefore be broadly applied in plant breeding.

## Author Contributions

VM and DG conceived a plan and draft of the manuscript, wrote the manuscript and gave final approval for submission.

## Conflict of Interest Statement

The authors declare that the research was conducted in the absence of any commercial or financial relationships that could be construed as a potential conflict of interest.

## Footnotes

1 http://mvd.iaea.org

## ABBREVIATIONS

**General abbreviations:**
AP: Apurinic/apyrimidinic
BER: Base excision repair
BLM: Bleomycin
Cas: CRISPR-associated
CPD: Cyclobutane pyrimidine dimer
CRISPR: Clustered Regularly Interspaced Short Palindromic Repeats
DASH: *Drosophila, Arabidopsis, Synechocystis*, Human
diRNA: DSB-induced small RNA
DSB: Double-strand break
EMS: Ethyl methanesulfonate
HR: Homologous recombination
IR: Ionizing radiation
LTRs: Long terminal repeats
miRNA: MicroRNA
MMR: Mismatch repair
MMS: Methyl methanesulfonate
NER: Nucleotide excision repair
NHEJ: Non-homologous end-joining
aNHEJ: Alternative DNA-PKcs-independent NHEJ
cNHEJ: Classical DNA-PKcs-dependent NHEJ
6-4 PP: Pyrimidine6-4pyrimidone photoproduct
REs: Restriction endonucleases
RNAi: RNA interference
RISC: RNA-induced silencing complex
ROS: Reactive oxygen species
SDSA: Synthesis-dependent strand annealing
SSA: Single-strand annealing
SSB: Single-strand break
siRNA: Small interfering RNA
ta-siRNA: Trans-acting siRNA
TALENs: Transcription Activator-Like Effector Nucleases
UV: Ultraviolet radiation
V(D)J recombination: Antigen receptor gene rearrangement of variable (V), diversity (D) and joining (J) gene segments
ZFNs: Zinc Finger Nucleases
**Gene and protein abbreviations:**
AGT: Alkylguanine DNA alkyltransferase
APLF: Aprataxin and PNK-like Factor
AGO: Argonaute
ARP: Apurinic endonuclease-redox protein
ASF: Anti-silencing function1
ATM: Ataxia-Telangiectasia mutated
ATR: ATM and RAD3-related
BRCA2: Breast Cancer2
CDK7: Cyclin-dependent kinase7
CEN2: Centrin2
COP1: Constitutive photomorphogenesis protein 1
CSA/B: Cockayne syndrome A/B
CSA-like: Cockayne syndrome-like
CUL4: Cullin4
DCL: DICER-like
DDB1/2: Damaged DNA binding1/2
DET1: De-etiolated1
DNA-PKcs kinase: DNA-Dependent Protein Kinase catalytic subunit
DOT1L: DOT1-like histone H3K79 methyltransferase
E2Fe/DEL1: atypical E2F transcription factor DP-E2F-like1
ELL: Eleven-nineteen lysine-rich leukemia (Elongation factor RNA polymerase II)
ERCC1: Excision Repair Cross-Complementation Group 1
EXO1: Exonuclease1
FACT: Facilitates chromatin transcription
FAD2: Fatty acid desaturation 2
FANCM: Fanconi anemia complementation group M
FEN1: Fla structure-specific endonuclease 1
FPG: Formamidopyrimidine-DNA glycosylase
GTF2H2: General transcription factor IIH, polypeptide 2
H: Histone
H2AX: Variant histone H2A
γH2AX: gamma-H2AX (phosphorylated H2AX)
HAT p300: Histone acetyltransferase p300
HIRA: Histone Cell Cycle Regulator
HMGN1: High Mobility Group Nucleosome Binding Domain 1
HR23B: UV excision repair protein RAD23 homolog B
HY5: Elongated hypocotyl5
HYH: HY5-homolog
KU70: X-ray cross complementing 6
KU80: X-ray cross complementing 5
LIG1: Ligase 1
LIGIV: Ligase 4
MRE11: Meiotic Recombination 11
MUS81: MMS and UV-sensitive protein 81 (Crossover junction endonuclease)
MutS/L: Mutator S/L
MLH: MutL homolog
MSH: MutS homolog
NBS1: Nijmegen breakage syndrome1
OGG1: Oxoguanine glycosylase
p53: Tumor protein 53
PARP: Poly (ADP-ribose) Polymerase
PCNA: Proliferating cell nuclear antigen
PHR1: CPD photolyase gene
PMS2: Postmeiotic Segregation Increased 2 (DNA Mismatch Repair Protein)
POLβ: DNA polymerase beta
Polymerase δ: DNA polymerase delta
POL: DNA polymerase lambda
RAD: Radiation sensitive
RecA: Recombination A
RECQ: ATP-dependent DNA helicase RecQ
RECQ4A: ATP-dependent DNA helicase Q-like 4A
RPII: RNA polymerase II
RPA: Replication protein A
RFC: Replication factor C
ROS1: Repressor of silencing1
SOG1: Suppressor of gamma response 1
SWI2/SNF2: SWItch/Sucrose Non-Fermentable
TFB1A: General transcription and DNA repair factor IIH B subunit 1-1
TFB5: General transcription factor IIH, polypeptide 5
TFIIH: Transcription factor II Human
TFIIS: Transcript elongation factor IIS
TTDA: Trichothiodystrophy group A
UVH6: Ultraviolet hypersensitive 6
UVR3: 6-4 photolyase gene
UVR8: UV resistance locus8
XRCC: X-ray repair cross complementing
XAB2: XPA binding protein 2
XLF: XRCC4-like factor
XPA-G: Xeroderma pigmentosum complementation group A-G
ZDP: Zinc finger DNA 3′-phosphoesterase
